# Food in the Reign of Henry the Seventh

**Published:** 1869-08

**Authors:** 


Food in the Reign of Henry the Seventh.—From Midsummer to Michaelmas was the only time they indulged in fresh meat, and the instructions say: "My lord has on his table, for breakfast, at seven in the morning, a quart of beer and wine, two pieces of salt fish, six red herrings, four white ones, and on flesh days, half a chine of beef or mutton boiled." At dinner, men ranking as knights had a table cloth, which was washed once a month; they had no napkins, and the fingers were extensively used in feeding.

